# Food and Medicine Homologous Plants in Osteoporosis: A Scoping Review of Preclinical Evidence

**DOI:** 10.1002/fsn3.71929

**Published:** 2026-06-08

**Authors:** Long Zhao, Xiao‐li Dong, Rui‐ting Li, Qu‐huan Ma, Xiao‐feng Shi

**Affiliations:** ^1^ College of Pharmacy Gansu University of Traditional Chinese Medicine Lanzhou China; ^2^ Pharmaceutical Research Institute of Gansu Academy of Medical Sciences Lanzhou China

**Keywords:** estrogen, food and medicine homology, osteoporosis, oxidative stress

## Abstract

Osteoporosis is a chronic systemic bone disease characterized by reduced bone density and an increased risk of fractures. This review summarizes the preclinical evidence for 61 FMH plants and their active ingredients. Its bone protection mechanism involves estrogen‐like action, inhibition of oxidative stress, regulation of intestinal flora and metabolites, regulation of autophagy, and other pathways. Based on a tertiary level of evidence, 24 plants had only a single animal/in vitro study (level 1), 34 had consistent results from multiple independent animal studies (level 2), and a few had preliminary human trials with obvious limitations (level 3). The existing evidence does not support FMH to replace standard anti‐osteoporotic drugs and should be positioned as an adjunctive strategy for long‐term prevention and early intervention, with standardized extracts and clinical trials with fractures as the endpoint in the future.

## Graphical abstract

Food and medicine homologous (FMH) plants and their bioactive compounds exhibit potential osteoprotective effects through multiple complementary mechanisms, including estrogen‐like signaling, attenuation of oxidative stress, modulation of the gut microbiota–bone axis, and regulation of autophagy. Representative FMH resources such as soybean, olive, ginger, *Lycium barbarum* , sesame, and Epimedium may collectively suppress osteoclastogenesis and bone resorption while promoting osteoblast differentiation, bone mineral density, and bone microarchitecture. Current evidence is predominantly derived from preclinical studies, whereas clinical evidence remains limited. Therefore, FMH resources should currently be considered supportive strategies for long‐term prevention and early intervention in osteoporosis rather than replacements for standard anti‐osteoporotic therapies.

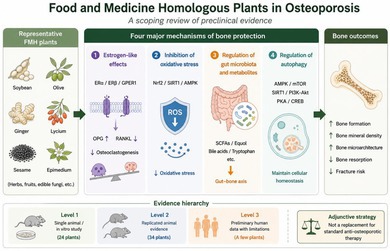

## 1 Introduction

Osteoporosis is a systemic bone disease characterized by decreased bone mass and disruption of bone microstructure, leading to increased bone fragility and an increased risk of fractures (Compston et al. 2019). According to the International Osteoporosis Foundation (2025) (IOF) report, the total number of osteoporosis patients worldwide has exceeded 500 million, and about one‐third of postmenopausal women suffer from the disease (International Osteoporosis Foundation 2025). The prevalence of postmenopausal women in China is about 26%, women over 50 years old reach 32.1%, and women over 65 years old rise to 51.6%. Approximately 210 million women nationwide are or are about to enter perimenopause, during which the average annual bone loss rate can reach 5% (Riggs et al. 2025). With the aging of the population, the burden of osteoporosis‐related fractures will continue to rise, and effective prevention and intervention strategies are urgently needed. Although commonly used clinical anti‐osteoporotic drugs such as bisphosphonates, selective estrogen receptor modulators, denosumab, and parathyroid hormone analogues can reduce the risk of fractures, there are problems such as atypical femoral fractures, osteonecrosis of the jaw, rebound bone loss after discontinuation, and long‐term compliance (Alnajmi et al. 2024; Walker and Shane 2023). Therefore, it is clinically important to explore new intervention strategies.

Epidemiological studies have linked specific dietary patterns to osteoporosis risk. A traditional Mediterranean diet rich in olive oil, fish, nuts, fruits, and vegetables is associated with higher bone density and a lower risk of hip fractures in older adults, and its polyphenols and omega‐3 fatty acids have anti‐inflammatory and antioxidant effects (Malmir et al. 2023; Noori et al. 2022). A traditional Japanese diet rich in soy isoflavones, seaweed, and fish can prevent bone loss in postmenopausal women, and prospective cohort studies have shown that natto intake is negatively correlated with fracture risk, and this association is independent of bone mineral density (Kojima et al. 2020). Conversely, Western diets high in red meat, processed meats, refined grains, sugary drinks, high‐fat dairy products, and low in fiber are positively associated with an increased risk of bone loss and fractures, possibly due to their pro‐inflammatory and acid‐loading effects (Denova‐Gutiérrez et al. 2018; Fabiani et al. 2019). It is worth noting that the key ingredients in the above‐mentioned beneficial diet come from olives, soybeans, and green tea, which all belong to the category of “medicine and food homology” (FMH) resources recognized by traditional Chinese medicine and national regulatory frameworks, which provides an epidemiological and ethnopharmacological basis for systematic research on FMH as a preventive or adjuvant treatment strategy for osteoporosis.

Despite the increasing attention to the control of osteoporosis in FMH plants, systematic reviews are still relatively lacking. This paper summarizes the main pathogenic mechanisms of osteoporosis in a structured manner and systematically expounds the preclinical evidence that FMH active ingredients exert osteoprotective effects, aiming to provide evidence‐based evidence for the prevention and treatment of osteoporosis and the development of functional products in ethnic medicine.

## 2 Main Factors and Signaling Pathways Influencing Osteoporosis

### 2.1 Estrogen

The core cause of postmenopausal osteoporosis is estrogen deficiency (Chen et al. 2022). Estrogen acts through the nuclear receptors ERα, ERβ, and membrane receptor GPER1 (Toumba et al. 2024). ERα is the main isoform expressed in osteoblasts, osteocytes, and osteoclast precursors (Zhivodernikov et al. 2023): in osteoblasts, ERα activation promotes proliferation, survival, and the expression of the osteogenic gene Runx2; in osteoclast precursors, ERα regulates the transcription of OPG and RANKL by inhibiting AP‐1/c‐Jun and stabilizing IκBα, inhibiting osteoclast production (Cheng et al. 2022). ERβ showed more tissue selectivity, mainly restricting osteoclast differentiation and regulating osteocyte function, while its direct effect on osteoblast proliferation was weaker. GPER1 further enables rapid non‐genomic activation of the PI3K/Akt and MAPK cascades, linking estrogen signaling to Wnt/β‐catenin stability and anti‐apoptotic pathways in osteoblasts and osteocytes (Luo et al. 2023).

In the RANKL/RANK/OPG system, the RANKL secreted by osteoblasts binds to the RANK on the surface of the osteoclast precursor to induce osteoclast differentiation. OPG neutralizes RANKL as a decoy receptor (Florencio‐Silva et al. 2022). Estrogen upregulates OPG through ERα and inhibits RANKL transcription, thereby inhibiting bone resorption. RANKL simultaneously activates the NF‐κB pathway, promotes the expression of c‐Fos and NFATc1, and drives osteoclast differentiation. Estrogen inhibits this pathway by increasing IκBα stability and interfering with the NF‐κB complex (Cheng et al. 2022). The isoflavones in soybean ( *Glycine max* ) need to be metabolized by the gut microbiota to equol, which acts as a highly selective ERβ agonist and inhibits osteoclast differentiation mainly by up‐regulating the OPG/RANKL ratio, while inhibiting RANKL‐dependent NF‐κB and NFATc1 signaling, thereby reducing bone resorption (Corbi et al. 2023; Sekikawa et al. 2025). Clinical studies have shown that equinol can reduce bone resorption markers (Taku et al. 2012), reflecting the leading role of ERβ in limiting osteoclast differentiation.

Wnt signaling promotes β‐catenin nuclear translocation and activates the osteogenic gene Runx2 (Florencio‐Silva et al. 2022). Estrogen enhances Wnt signaling by downregulating SOST and DKK1 and inhibiting GSK3β activity. *Puerarin in Pueraria montana* activates both ERα and ERβ and stimulates osteoblast differentiation and bone formation through p38 MAPK and Wnt/β‐catenin pathways. Its regulation of OPG and RANKL is co‐mediated by two ER isoforms, while the inhibition of IL‐6 is mainly achieved through ERα. Puerarin has a much weaker stimulating effect on mammary epithelial cells than estrogen, showing tissue‐selective safety characteristics (Wang and Li 2013).

**FIGURE 1:**
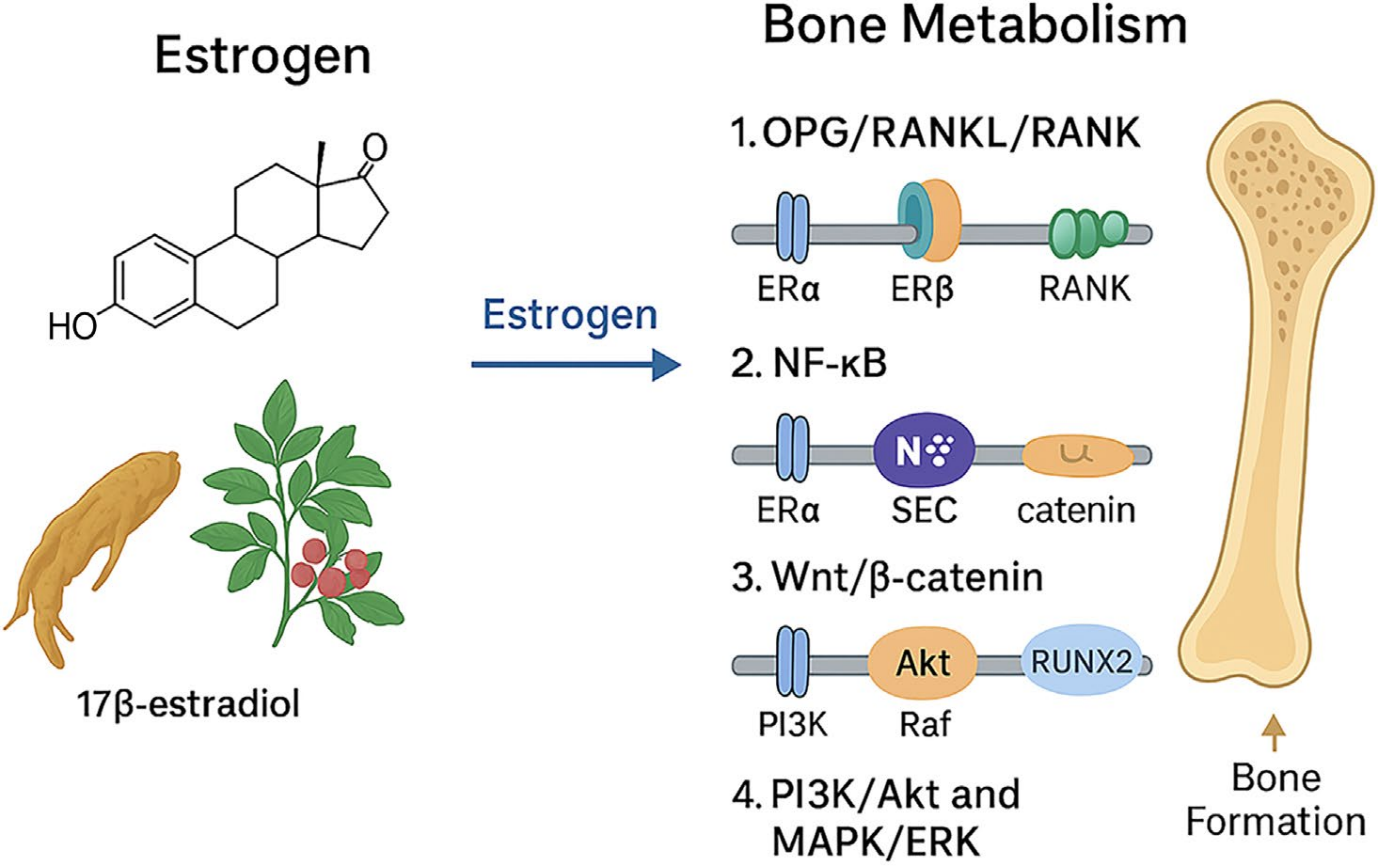
Effects of estrogen on bone formation.

In addition, estrogen promotes osteoblast survival through GPER1 activation of PI3K/Akt, while activation of MAPK/ERK enhances Runx2 activity and osteogenic differentiation. *Icariin in Epimedium brevicornum* does not directly bind to the ER ligand‐binding domain, but rapidly activates the MAPK/ERK and PI3K/Akt pathways through non‐genomic pathways, inducing ERα phosphorylation at Ser118 and Ser167 sites, thereby promoting osteoblast proliferation, differentiation, and survival (Song et al. 2020). This process simultaneously enhanced the cross‐dialogue between IGF‐1 signaling and ERα, significantly improving bone loss in OVX rats without causing uterine hyperplasia (Zou et al. 2024), demonstrating the dominant role of ERα in promoting bone formation. FMH regulates bone metabolism by regulating the above pathways through estrogen‐like effects (Figure 1).

### 2.2 Oxidative Stress

**FIGURE 2:**
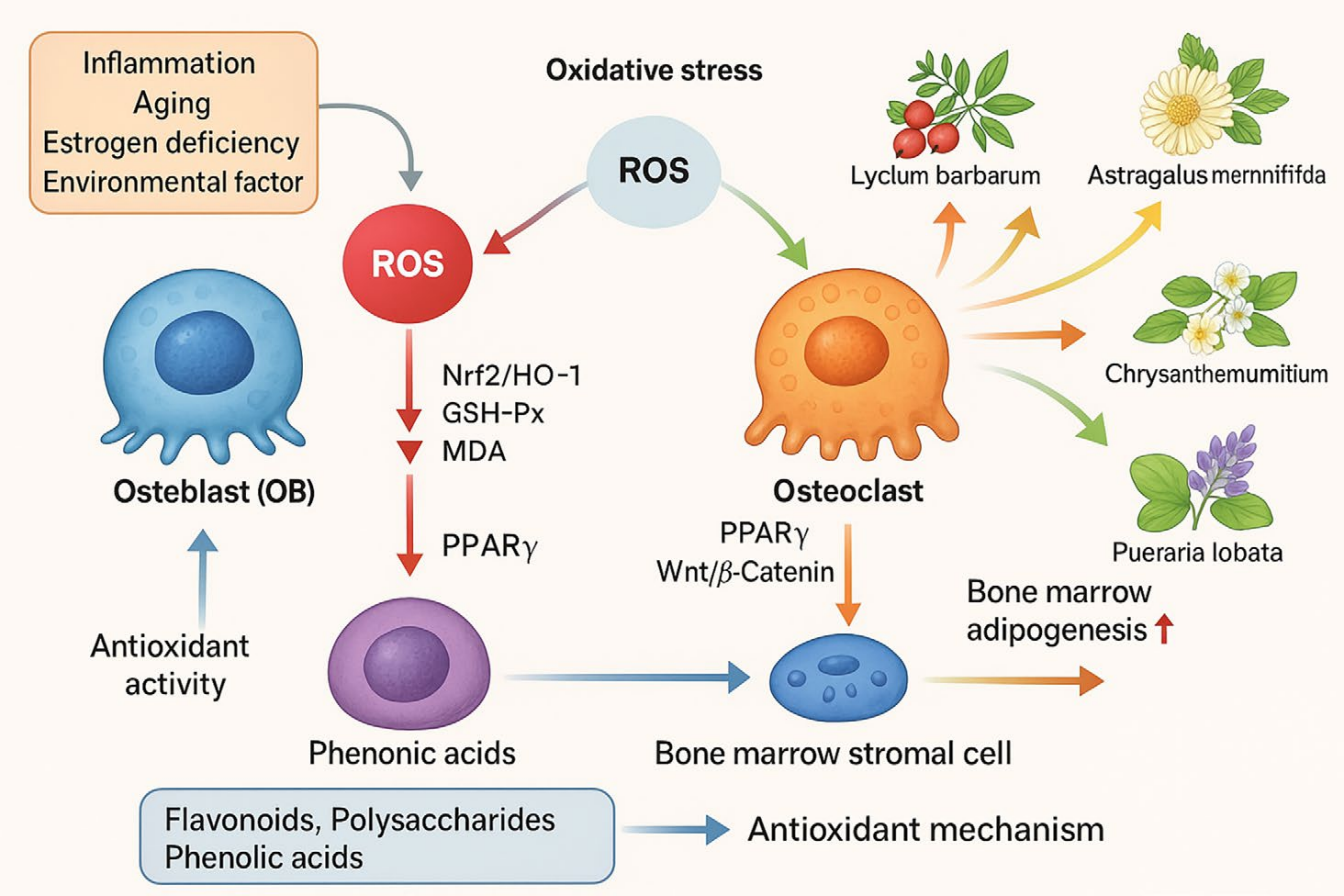
Effects of oxidative stress on osteoblasts and osteoclasts.

Oxidative stress (OS) is a pathological condition in which reactive oxygen species (ROS) produce more than endogenous antioxidant defenses. In bone, ROS is mainly derived from the mitochondrial electron transport chain and the NADPH oxidase (NOX) family. Moderate ROS is involved in bone metabolism as a signaling molecule, but excess ROS can lead to bone remodeling imbalance: inhibiting osteoblast activity and promoting osteoclast production is a key molecular mechanism in postmenopausal and senile osteoporosis (Marques‐Carvalho et al. 2023). Excess ROS promotes β‐catenin degradation by activating GSK3β, inhibits Wnt/β‐catenin and BMP/Smad pathways, and reduces Runx2 and Osterix expression, thereby inhibiting osteogenic differentiation and mineralization. At the same time, ROS induces endoplasmic reticulum stress and mitochondrial dysfunction, leading to osteoblast apoptosis (Liu, Shen, et al. 2024). ROS acts as a second messenger to enhance RANKL‐RANK‐TRAF6 signaling, activate NF‐κB and MAPK pathways, and promote NFATc1 nuclear translocation, thereby driving osteoclast differentiation. In addition, ROS stimulates RANKL secretion and downregulates OPG, further enhancing bone resorption. FMH protects bones primarily through the following antioxidant defense pathways (Figure 2).

#### 2.2.1 Nuclear Factor Erythroid 2‐Related Factor 2 (Nrf2) Signaling Pathway

Nrf2 is a central transcription factor in antioxidant responses (Choi et al. 2023). In physiological conditions, Nrf2 activation can induce antioxidant enzymes such as HO‐1 and NQO1 to protect bone cells. However, under chronic or severe oxidative stress, excess ROS can damage the Nrf2 nuclear translocation and promote its degradation, leading to a decrease in antioxidant capacity and a vicious cycle (Marques‐Carvalho et al. 2023).

The active ingredient in medicinal and food homologous plants effectively activates the Nrf2 pathway. For example, curcumin (derived from *the rhizome of Curcuma longa*) maintains the stability of Nrf2 by inhibiting GSK3β activity, preventing GSK3β‐mediated phosphorylation of serine (Ser40) at the 40th position of Nrf2, promoting the dissociation of Nrf2 from Keap1 and translocation to the nucleus, thereby inducing the expression of antioxidant enzymes such as HO‐1, NQO1, and GCLM, clearing ROS, and protecting osteoblasts (Li 2022). In addition, curcumin inhibits RANKL‐mediated NF‐κB signaling by blocking IκBα phosphorylation and p65 nuclear translocation, downregulates NFATc1 and c‐Fos, and reduces the expression of osteoclast‐specific genes TRAP, CTSK, and MMP‐9 (Ding et al. 2024).

#### 2.2.2 SIRT1/FOXO3a Pathway

SIRT1 reduces ROS damage by deacetylating FOXO3a, activating antioxidant genes such as MnSOD and CAT. Oxidative stress can reduce SIRT1 activity, weakening this protective mechanism (Kitada et al. 2024). Resveratrol, which is present in grapes, berries, and some medicinal plants, is a highly potent SIRT1 activator (Hairi et al. 2023). It deacetylated FOXO3a by activating SIRT1 and upregulated the expression of MnSOD and CAT. At the same time, Nrf2 nuclear translocation and ARE activity were enhanced, and endogenous antioxidant capacity was synergisively enhanced. Resveratrol also inhibited RANKL‐induced osteoclast differentiation by inhibiting IκBα phosphorylation and NF‐κB p65 nuclear translocation, and inhibiting the production of inflammatory factors TNF‐α, IL‐6, and IL‐1β. In terms of osteogenesis, activation of the Wnt/β‐catenin pathway enhances β‐catenin nuclear transposition, upregulates Runx2 and Osterix, and promotes osteoblast survival through the PI3K/AKT pathway (Feng et al. 2024).

In addition, *Lycium barbarum* polysaccharide (LBP) also inhibited osteoblast apoptosis by activating the SIRT1/FOXO3a pathway. LBP up‐regulated SIRT1 expression and deacetylase activity, deacetylated FOXO3a translocated to the nucleus, transcriptionally activated antioxidant and DNA repair genes such as MnSOD, CAT, and GADD45, and reduced ROS levels. At the same time, it reduces the Bax/Bcl‐2 ratio, inhibits caspase‐3/9 activation, inhibits the mitochondrial apoptosis pathway, and protects osteoblasts. Studies have shown that LBP promotes the proliferation, migration, and differentiation of osteoblasts while inhibiting osteoclast production and promoting their apoptosis (Li et al. 2023).

#### 2.2.3 AMPK Pathway

AMPK is a key regulator of energy metabolism and mitochondrial homeostasis, promoting mitochondrial biosynthesis and inhibiting ROS production by activating PGC‐1α/TFAM. Persistent oxidative stress inhibits AMPK activity and exacerbates mitochondrial dysfunction (Khalil et al. 2022).

A variety of active ingredients in medicinal and food homologous plants exert mitochondrial protection and antioxidant effects by activating the AMPK pathway. As previously mentioned, resveratrol can enhance PGC‐1α/TFAM signaling by activating AMPK, promote mitochondrial biosynthesis, improve mitochondrial function, and alleviate oxidative stress at the metabolic level (Zhou et al. 2023). In addition, ginsenoside Rg3 (derived from *Panax ginseng* ) promotes autophagy by modulating the AMPK/mTOR signaling pathway, improving bone density and improving bone microstructure in aged osteoporosis rats (Zhang et al. 2024). Icariin (derived from *Epimedium brevicornum*) can inhibit mTOR phosphorylation and promote the level of autophagy in bone marrow mesenchymal stem cells by upregulating AMPK phosphorylation levels, thereby promoting its osteogenic differentiation, inhibiting lipogenic differentiation, and improving bone mass in castrated osteoporosis mice (Chen et al. 2023).

#### 2.2.4 NF‐κB Pathway

NF‐κB signaling pathway is a key inflammatory pathway that mediates osteoclast differentiation, and inhibition of this pathway is also one of the important antioxidant mechanisms of FMH active ingredients. Curcumin inhibits RANKL‐mediated NF‐κB signaling by blocking IκBα phosphorylation and p65 nuclear translocation (Jantarawong et al. 2023). Puerarin (derived from *Pueraria montana* ) maintains bone mass by inhibiting the activation of NF‐κB and MAPK signaling pathways, while stimulating the expression of OPG in osteoblasts and inhibiting the production of RANKL and IL‐6 (Zhao et al. 2024; Xiao et al. 2023). Quercetin can inhibit the NF‐κB inflammatory pathway, and at the same time, it achieves “bidirectional regulation” at the molecular level by regulating the AMPK/SIRT1 axis and activating the Wnt/β‐catenin pathway, which not only inhibits bone resorption but also promotes bone formation (Feng et al. 2023). Medicinal and edible homologous plants rich in quercetin, rutin, and kaempferol include 20 species of plants such as locust rice, mulberry, hawthorn, chrysanthemum, honeysuckle, peppermint, sea buckthorn, wolfberry, and red dates, which can exert anti‐osteoporosis effects by inhibiting the NF‐κB signaling pathway and other mechanisms (Xiong et al. 2021).

In summary, the above‐mentioned FMH active ingredients form a multi‐layered antioxidant defense network from different perspectives, such as activating Nrf2/HO‐1, SIRT1/FOXO3a, AMPK, and inhibiting NF‐κB, and synergistically protect bone cells from oxidative damage (Figure 2).

### 2.3 Gut Microbiota

The gut microbiota is involved in the regulation of bone homeostasis through a variety of pathways. Dysbacteriosis caused by antibiotics, oophorectomy, high‐fat, low‐fiber diet, and so forth, can lead to increased intestinal permeability, endotoxin (LPS) entering the bloodstream, activating systemic inflammatory responses, and stimulating osteoclast production.

#### 2.3.1 Short‐Chain Fatty Acids (SCFAs)

Intestinal flora ferments dietary fiber to produce short‐chain fatty acids (SCFAs), mainly including acetic acid, propionic acid, and butyric acid. SCFAs promote regulatory T cell accumulation, inhibit RANKL expression, and reduce osteoclast formation by activating GPR41/GPR43 receptors and inhibiting histone deacetylase (HDAC) (Li et al. 2024). SCFAs also directly regulate the metabolism of osteoclast precursors. Polysaccharide components can play a bone‐protective role by enriching SCFA‐producing bacteria, *and astragalus polysaccharides in Astragalus mongholic can* significantly change the structure of intestinal flora, enrich SCFA‐producing bacteria such as Ruminococcaceae, Blautia, and Lactobacillus, increase the levels of acetic acid, propionic acid, and butyric acid, and promote Treg differentiation. Inhibition of RANKL‐induced osteoclast production (Li 2022). *Eucommia ulmoides* leaf extract can promote the growth of the probiotic *Lactobacillus bulgaricus* , increase intestinal microbiota diversity and Firmicutes/Bacteroidetes ratio, and increase the concentration of SCFAs in feces and serum (especially butyric acid), thereby improving bone microstructure, increasing bone density, and inhibiting osteoclast production (Xie et al. 2023; Yin et al. 2025). *Cistanche deserticola* extract regulates intestinal microbiota disorders, promotes the proliferation of SCFA‐producing bacteria such as Firmicutes, Roseburia, and Oscillibacter, and enhances intestinal barrier function, thereby protecting OVX mice from bone loss (Yang et al. 2023).

#### 2.3.2 Phytoestrogen Metabolism

The gut microbiota converts soy isoflavones (such as lignin) to equol, a highly selective ERβ agonist with activity that inhibits bone resorption (Yang et al. 2013). Differences in conversion capacity between individuals explain the heterogeneity in efficacy in some clinical studies (Leonard et al. 2024). The isoflavones (including puerarin, daidzein, and lignin) in *Pueraria montana* need to be hydrolyzed into the form of glycosides (daidzein, lignin) by the intestinal flora before they can be absorbed and further metabolized into active metabolites such as equinol (Rizzo and Baroni 2022). Pueraria kudzu extract could significantly increase the production of equinol in vitro fermentation system, and the abundance of equinol‐producing bacteria (such as Eggerthella, Adlercreutzia) was positively correlated with the osteoprotective effect. In addition, the flavonoids in hawthorn ( *Crataegus pinnatifida* ) can promote the aqueous release of isoflavone glycosides in the diet by regulating the activity of β‐glucosidase in the gut microbiota, thereby indirectly enhancing the biotransformation of phytoestrogens and their osteoprotective effects (Zhou et al. 2022).

#### 2.3.3 Tryptophan/Bile Acid/Serotonin Metabolic Pathway

**FIGURE 3:**
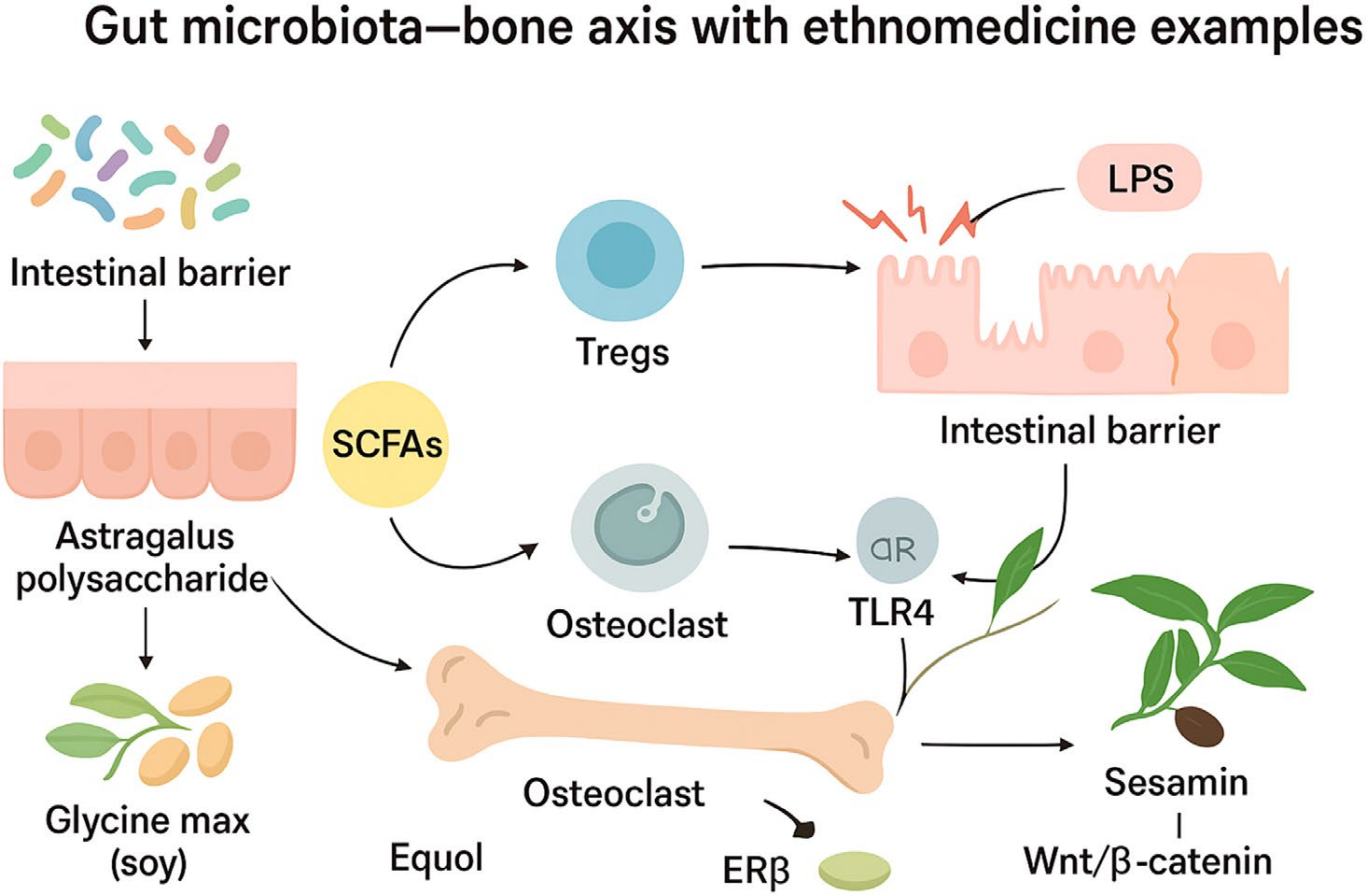
Gut microbiota‐bone axis with ethnomedicine examples.

Tryptophan metabolites (indoles) regulate bone immunity through aromatic hydrocarbon receptors (AhR), secondary bile acids affect mesenchymal stem cell osteogenic differentiation through FXR and TGR5 signaling, and gut‐derived serotonin inhibits bone formation through LRP5 (Yadav and Ducy 2023; Xiang et al. 2025). Triterpenoids and polysaccharides in *Ganoderma lucidum* regulate bile acid metabolism, increase the level of secondary bile acids (e.g., deoxycholic acid, lithocholic acid), and promote the differentiation of bone marrow mesenchymal stem cells into osteoblasts through FXR/TGR5 signaling (Grienke et al. 2023). The triterpenoids in *Poria cocos* can inhibit intestinal serotonin synthesis, reduce serum serotonin levels, relieve its inhibition of LRP5‐mediated bone formation, and increase trabecular bone volume in OVX rats (Yadav and Ducy 2023). The catechin EGCG in green tea ( *Camellia sinensis* ) modulates tryptophan metabolic pathways, reduces the production of the inflammatory metabolite quinoline acid, while enhancing AhR‐mediated anti‐inflammatory responses and indirectly exerts osteoprotective effects (Reynolds and Brown 2024). In addition, quinoa ( *Chenopodium quinoa* ) repairs the intestinal barrier by upregulating the expression of tight junction proteins (occludin, claudin‐1, and ZO‐1) in the duodenum, reversing dysbacteriosis (increasing Firmicutes, decreasing Bacteroidetes and Prevotella), and improving bone microstructure and bone metabolism (Pan et al. 2025). In summary, the gut microbiota is an important mediator of osteoprotective role in medicinal and food homologous plants, and Figure 3 shows the relevant mechanisms, involving the SCFA‐Treg‐RANKL axis, the phytoestrogens‐equineol‐ERβ axis, and the tryptophan/bile acid/serotonin metabolism pathway.

### 2.4 Autophagy

Autophagy disorders are an important mechanism of osteoporosis and manifest as bidirectional regulation in osteocytes. Basal or moderately induced autophagy (via AMPK activation and moderate mTOR inhibition) promotes mitochondrial quality control, induces ROS reduction through SIRT1/FOXO3a and ATG, and promotes osteoblast survival and differentiation. However, persistent stress, severe endoplasmic reticulum stress, or prolonged Akt inhibition can lead to excessive autophagy activation, triggering autophagy‐dependent cell death and making cells more sensitive to caspase‐mediated apoptosis (Zhao et al. 2025). The molecular mechanisms involved include upregulation of Beclin‐1/LC3, changes in p62/SQSTM1 accumulation kinetics, and interactions with Bcl‐2 family apoptosis regulators. Therefore, therapeutic interventions should aim to restore physiological autophagy flux rather than indiscriminately activate autophagy (Zhao et al. 2023).

#### 2.4.1 AMPK/mTOR

AMPK activation inhibits mTORC1, which induces autophagy, helps osteoblasts survive under stress conditions, and promotes differentiation. *After* treatment of MC3T3‐E1 osteoblasts with strychanoside (10, 20 μmol/L), the AMPK phosphorylation level was significantly increased, the mTOR phosphorylation level decreased, the LC3‐II/LC3‐I ratio increased, and the number of autophagosomes increased after treatment with strychonin (10, 20 μmol/L). These effects can be reversed with the AMPK inhibitor Compound C. In the OVX rat model, strychanoside (20 mg/kg/day for 12 weeks) significantly increased trabecular volume fraction and bone density, promoting the expression of osteogenic markers (Park et al. 2021). In addition, sesamin in black sesame seeds ( *Sesamum indicum* ) also enhances autophagy through the AMPK/mTOR pathway, protects osteoblasts from oxidative stress‐induced apoptosis, and promotes bone formation (Cai et al. 2022).

#### 2.4.2 PI3K/Akt/mTOR

Akt inhibition overactivates autophagy, which may damage cells; moderate autophagy has a protective effect on osteoblasts. The active ingredients in medicinal and food homologous plants can restore autophagy balance by moderately regulating this pathway. Isoliquiritigenin in Glycyrrhiza uralensis regulates autophagy bidirectionally through the PI3K/Akt/mTOR signaling pathway (Zhu et al. 2021). In osteoblasts, isoglycyrritigenin (1–10 μmol/L) moderately inhibits Akt phosphorylation, restores autophagy flux, and protects cells from glucocorticoid‐induced apoptosis; in osteoclast precursors, isoglycyrritigenin inhibits RANKL‐induced overexpression of Beclin‐1 and LC3‐II and reduces osteoclast differentiation. In vivo studies have confirmed that isoliquiritigenin (50 mg/kg/day for 8 weeks) improves bone microstructure and increases bone density in OVX rats without causing uterine hyperplasia.

#### 2.4.3 SIRT1/FOXO3a

SIRT1 activation deacetylates FOXO3a, upregulates autophagy‐related genes (ATG5, ATG7), and promotes osteoblast survival. The active ingredient in medicinal and food homologous plants enhances autophagy by activating this axis. The polysaccharides in Polygonatum sibiricum promote autophagy by activating the SIRT1/FOXO3a pathway (Gong et al. 2021). After gavage administration of glucocorticoid‐induced osteoporosis rats with glucocorticoids, the expression of SIRT1 and strainylase activity in bone tissue was significantly increased, FOXO3a was translocated to the nucleus after deacetylation, the ratio of LC3‐II/LC3‐I increased, and the expression of p62 decreased, while the Bax/Bcl‐2 ratio, caspase‐3 activation, and mitochondrial apoptosis pathway were inhibited. Thus protecting osteoblasts.

#### 2.4.4 PKA/CREB

**FIGURE 4:**
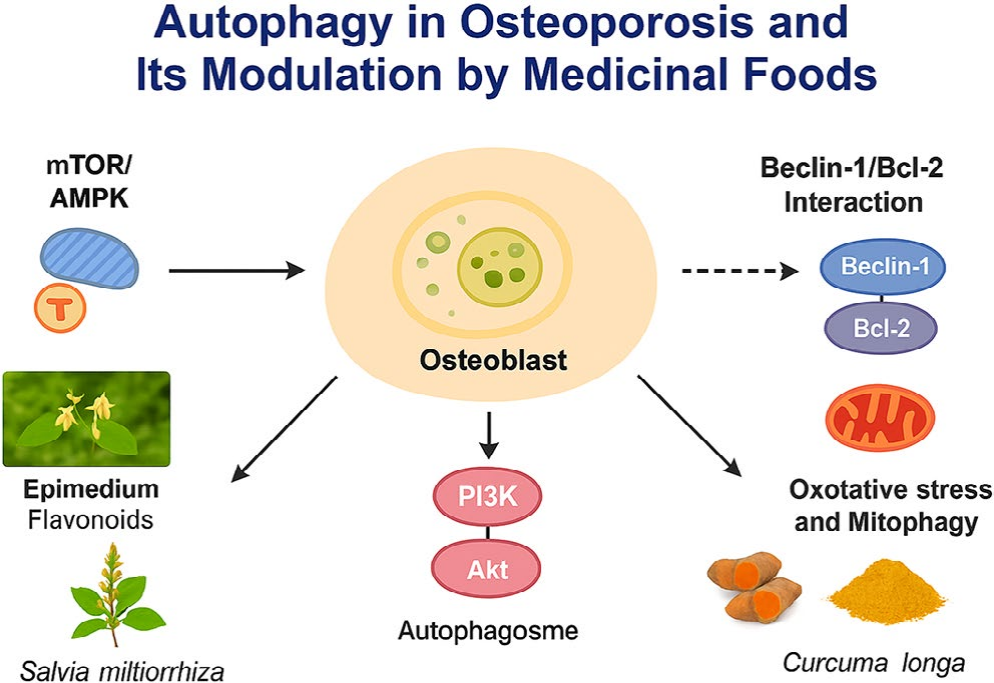
Autophagy in osteoporosis and its modulation by medicinal foods.

The cAMP/PKA signaling pathway can upregulate the expression of autophagy‐related genes through phosphorylation of CREB and induce autophagy. *Galangin in Alpinia officinarum* induces autophagy in bone marrow mesenchymal stem cells by activating the PKA/CREB signaling pathway, alleviating glucocorticoid‐induced osteoporosis. After treatment with galangal (5, 10, 20 μmol/L), the phosphorylation level of PKA increased, the transcriptional activity of CREB was enhanced, the expression of autophagy‐related genes ATG5 and LC3‐II was up‐regulated, and the autophagy flux was enhanced. In a rat model of glucocorticoid‐induced osteoporosis, galangin (40 mg/kg/day for 8 weeks) significantly increased bone volume fraction and trabecular thickness, reduced trabecular resolution, and up‐regulated the expression of p‐PKA, p‐CREB, and autophagy‐related proteins (Zeng et al. 2023). In summary, a variety of active ingredients in medicinal and food homologous plants moderately enhance autophagy in osteoblasts to promote bone formation and inhibit excessive autophagy in osteoclasts to reduce bone resorption by activating autophagy‐related signaling pathways such as AMPK/mTOR, PI3K/Akt/mTOR, SIRT1/FOXO3a, and PKA/CREB (Figure 4), respectively. Thus, physiological autophagy flux is restored and bone metabolic balance is regulated in both directions.

## 3 Methodology

**FIGURE 5:**
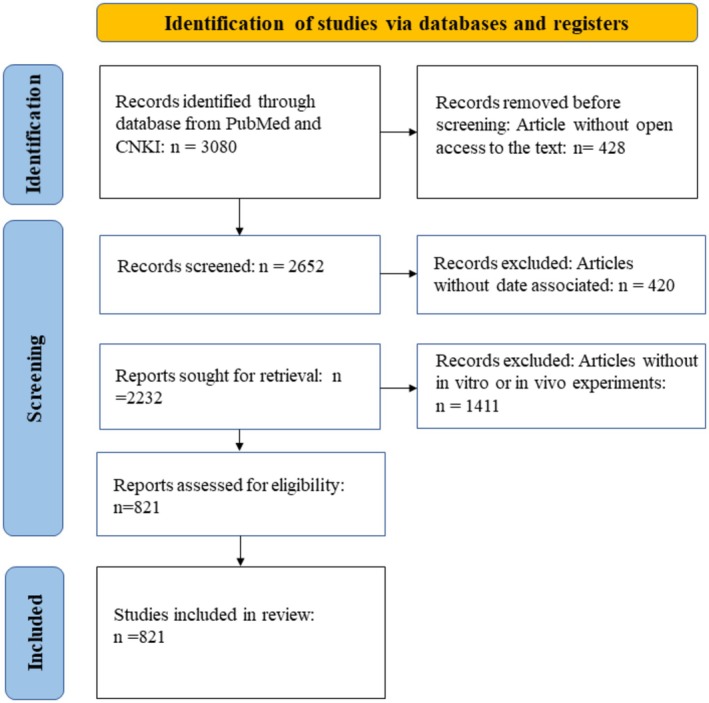
PRISMA flow diagram.

A scope review of the literature on the improvement of osteoporosis by medicinal and food homologous plants. Referenced databases include PubMed and CNKI. Articles were selected based on title, publication within 15 years, abstract review, and full‐text reading to assess their relevance. The search keywords include 106 plants in the list of medicinal and food homology released by the Chinese Health Commission, a combination of words such as “osteoporosis” and “bone tissue.” Articles that were not available in full, were not within 15 years, and did not include in vivo, in vitro, and randomized controlled trials were not included in the final analysis (see Figure 5).

## 4 Effects of Medicinal and Edible Plants on Osteoporosis

To systematically assess the level of available evidence for the anti‐osteoporosis effects of medicinal and food homologous plants, this study divided the supporting evidence for each substance (or its active ingredient) into the following three levels:

Level 1 (single animal studies or in vitro studies only): refers to reports of in vitro studies supported by only individual animal experiments, or based only on the cellular level (e.g., osteoblasts, osteoclasts, or bone marrow mesenchymal stem cells) without in vivo replicate validation.

Among the FMH plants belonging to this class, only single animal researchers included clove, cumin, bergamot, bitter almond, houttuynia, light bamboo leaf, dandelion, coix seed, and so forth, and their active ingredients showed osteoprotective effects in OVX or glucocorticoid‐induced osteoporosis models, but there was a lack of repeated verification by multiple models. Only in vitro cell experimenters include monk fruit, honeysuckle, hawthorn, chrysanthemum, perilla, peppermint, and so forth, whose extracts or active ingredients show activity in osteoblasts, osteoclasts, or mesenchymal stem cells to promote osteogenic differentiation or inhibit osteoclasts, but have not been proven at the animal level. In addition, longan meat and peppercorns have only been reported in non‐mammalian models such as zebrafish, and the strength of the evidence is also classified as this level (Table 1).

**TABLE 1:** Single animal study or in vitro only (level 1).

S. No.	Plants source	Active compound	Model	Dosage	Modulating effect	References
1	Myrtaceae, Syzygium	Ethanolic Extract of Clove Bud	OVX model	50% hydroalcoholic extract, 4 weeks	Increases BMD, BMC and bone tensile strength, exerting bone‐protective effects against hypogonadal osteoporosis	Karmakar et al. 2012
2	Apiaceae, Foeniculum	Fennel seed extract	OVX model	30, 100 mg/kg FvMs, 6 weeks	Attenuates OVX‐induced bone loss by mitigating estrogen‐dependent bone resorption and preventing the concomitant deterioration in biomechanical strength.	Kim et al. 2012
3	Rutaceae, Citrus	Bergapten	BMSCs, OVX model	In vitro: 0.1, 1, 10 μmol/L, 14 days, in vivo: 20 mg/kg/day, 2 months	Promotes osteogenic differentiation of BMSCs via activation of the Wnt/β‐catenin pathway; ameliorates osteoporosis in OVX mice by enhancing bone formation	Wang 2016
4	Rutaceae, Citrus	(same plant, different study)	OPG‐KO‐DO model	10, 20 mg/kg/day, 20 weeks	Ameliorates diabetes‐related osteoporosis by inhibiting PI3K/AKT, JNK/MAPK, and NF‐κB pathways, reducing osteoclastogenesis, and improving metabolic and inflammatory parameters	Li et al. 2016
5	Rosaceae, Prunus	Amygdalin (AD)	OVX model	0.1, 0.5, and 1.0 mg/kg/day, 90 days	Attenuates oxidative stress, promotes fracture healing, and alleviates osteoporotic symptoms in rats	Wang, Wang, et al. 2024
6	Cucurbitaceae, Siraitia	Mogroside V	T2D model, BMSCs	6.25 × 10^−3^ g/L, 7–14 days	Improves glucose metabolism, proliferation and anti‐inflammatory response	Luo et al. 2022
7	Caprifoliaceae, Lonicera	Mangiferin, chlorogenic acid	hMSCs cells, BMMs cells	1, 10 and 20 μg/mL, 48 h–18 days	Promotes osteogenesis and inhibits osteoclastogenesis	Seo et al. (2016)
8	Saururaceae, Houttuynia	Essential oil	OVX model	10, 20 mg/kg/day, 12 weeks	Attenuates OVX‐induced osteoporosis	Huang et al. 2019
9	Rubiaceae, Gardenia	Geniposide	HFD‐OP model	100 mg/kg, 6 months	Ameliorated high‐fat diet‐induced osteoporosis and OX‐LDL‐induced osteoblast apoptosis	Xiao et al. 2023
			MC3T3‐E1 cells			
10	Moraceae, Morus	Aqueous extract	HSHFD/STZ‐DM model	1, 2 g/kg/day, 12 weeks	Ameliorates bone loss and oxidative stress	Liu et al. 2021
11	Moraceae, Morus	Oligonucleotide ASON‐OP	OVX model	2 mg/(kg/BW/day), 4 weeks	Promotes osteogenesis and inhibits bone resorption, thereby ameliorating osteoporosis	Mu and Wang 2024
12	Moraceae, Morus	Quercetin, kaempferol, beta‐carotene, beta‐sitosterol, cyanin, morin	TM3 cells, MC3T3‐E1 cells	12.5, 25, 50, 100, 200 μg/mL, 24 h	Promotes osteoblast proliferation and differentiation, enhances testosterone secretion, and improves bone formation activity	Liu et al. 2021
13	Nelumbonaceae, Nelumbo	Polysaccharides from lotus leaves	OVX model, BMMs cells	30, 100 mg/kg/day, 4 weeks; 3.13–200 μg/mL, 4 days	Inhibits osteoclastogenesis and protected against estrogen deficiency‐induced bone loss by downregulating c‐Fos/NFATc1 signaling	He et al. 2021
14	Nelumbonaceae, Nelumbo	Alkaloids and flavonoids	OVX model	300 mg/kg, 6 weeks	Improves bone structure	Park et al. 2023
15	Poaceae, Lophatherum	Water extract	OVX model	100, 300 mg/kg/day, 6 weeks	Inhibits osteoclastogenesis, prevents bone loss	Lee et al. 2022
16	Asteraceae, Chrysanthemum	Ethyl acetate fraction, water fraction and compounds 5–11, 18, 19	RAW 264.7 cells	10 μmol/L, 5 days	Exhibits significant antioxidant activities and potent anti‐osteoporotic effects	Luyen et al. 2015
17	Lamiaceae, Perilla	Luteolin, baicalein, apigenin, kaempferol, quercetin, rosmarinic acid, rutin	RAW264.7 cells, MG‐63 and SAOS‐2 cells	12.5–50 μg/mL, 6 days; 12.5–50 μg/mL PLH for 72 h	Inhibits osteoporosis by reducing urinary calcium excretion and improving bone strength under food restriction	Phromnoi et al. 2022
18	Lamiaceae, Perilla	Perilla oil	50% FR‐OP rats	14%, 3 weeks	Ameliorates osteoporosis by promoting bone formation, inhibiting bone resorption, and modulating miRNA‐21‐5p/TGF‐β1/Smads signaling	Sun et al. 2004
19	Piperaceae, Piper	Piperine	Zebrafish embryos, Juvenile zebrafish, Adult zebrafish GIOP model	250 nmol/L, 3 days; 500 nmol/L, 14 days	Restores alveolar bone mass and microarchitecture in estrogen‐deficient mice	Carnovali et al. 2021
20	Asteraceae, Taraxacum	Chlorogenic acid, Caffeic acid, Luteolin 7‐O‐glucoside	OVX model	12.64, 78.36 mg/kg	Inhibits osteoclastogenesis and bone resorption via RANKL‐c‐Fos/NFATc1 pathway, protecting against OVX‐induced bone loss	Heo et al. 2022
21	Rhamnaceae, Ziziphus	Saponin A	MC3T3‐E1 cells	5, 10, 20, 40 mg/L	Promotes proliferation, reduces apoptosis, enhances differentiation and osteogenic efficiency possibly via TGF/BMP signaling	Zhang et al. 2022
22	Lamiaceae, Mentha	Ethanolic extract, aqueous extract	RAW 264.7 cells	1, 5, 10 μg/mL	Both extracts exhibited relatively strong inhibitory effects on TRAP activity and dose‐dependent suppression of osteoclast differentiation	Xu and Wang 2017
23	Poaceae, Coix	Aqueous extract of adlay seed	OVX model	0.3 g/kg	Enhances osteoblast proliferation through ERK signaling pathway	Yang et al. 2013
24	Iridaceae, Crocus	Crocin	OVX model	20 mg/kg	Inhibits osteoporosis development possibly by suppressing osteoblast apoptosis via PI3K/Akt signaling	He et al. 2021

In summary, a total of 24 FMH plants are currently at the level of Level 1 evidence, and their bone protection potential is worthy of attention, but more independent animal experiments and preclinical studies are urgently needed to verify it.

Level 2 refers to at least two independent animal studies with high methodological quality and low risk of bias that have consistently supported the plant's improvement in bone mineral density, bone microstructure, or markers of bone metabolism. This level represents reliable preclinical evidence. This level represents reliable preclinical evidence that at least two independent animal studies of high methodological quality have obtained consistent results supporting the plant's improvement in bone density, bone microstructure, or markers of bone metabolism. There are 34 FMH plants belonging to this class, and the evidence is consistent in vitro and in vitro, covering a variety of osteoporosis models and signaling pathways (Table 2).

**TABLE 2:** Multiple animal model studies (level 2).

S. No.	Plants source	Active compound	Model	Dosage	Modulating effect	References
1	Dioscoreaceae, Dioscorea	Chinese yam extract	OVX model	400 mg/kg/day, 14 weeks	Exhibits efficacy in regulating menopausal osteoporosis	Park and Kim 2024
2	Rosaceae, Crataegus	Chlorogenic acid	RAW 264.7 cells	125, 250, 500, 1000 μg/mL, 24 h	Inhibits osteoclast differentiation and prevent OVX‐induced bone loss, likely via downregulating osteoclastic transcription factors and genes	Ho et al. 2024
3	Portulacaceae, Portulaca	RANKL‐induced osteoclasts (EPE)	GIO Model	100 mg Dex/kg	Improves bone quality and histology by modulating bone formation, resorption, calcium homeostasis, and antioxidant defenses	Salekzamani et al. 2023
4	Fabaceae, Glycyrrhiza	18β‐Glycyrrhetinic acid	OVX model	50 mg/kg	Reduces bone loss in OVX mice by inhibiting osteoclastogenesis	Chen et al. 2018
		Glabridin	MC3T3‐E1 cells	1–10 μmol/L	Enhances osteoblast function	Choi et al. 2023
5	Apiaceae, Angelica	Angelica dahurica water extract	OVX model	100, 300 mg/kg/day	Ameliorates bone loss while improving weight gain and hepatic lipid accumulation in OVX, without estrogenic effects	Carnovali et al. 2019
6	Sapindaceae, Dimocarpus	*Dimocarpus longan* fruit extract	Zebrafish	LFE (6.25, 12.5, 25, 50, 100 ppm), 6 days	Promotes bone mineralization	Zhu and Wang 2012
7	Myristicaceae, Myristica	Macelignan (MRL)	OVX model	15, 30 mg/kg	Inhibits osteoclastogenesis and prevents bone loss via suppression of mitochondrial function and ERK signaling	Gu et al. 2023
8	Lauraceae, Cinnamomum	Cinnamic aldehyde	OVX model	75 mg/kg/day, 12 weeks	Ameliorates OVX‐induced bone loss by improving bone microarchitecture and density, and reducing pro‐inflammatory cytokines	Son and Lee 2019
		Macelignan (MRL)	HFD model	40 mg/kg/day, 8 weeks	Ameliorates HFD‐induced bone deterioration by improving bone microstructure, enhancing biomechanical strength, and downregulating Cathepsin K expression	Yang et al. 2023
9	Elaeagnaceae, Hippophae	Seabuckthorn polysaccharide	GIMD model	50, 100, 200 mg/kg/day, 4 weeks	Prevents and treats postmenopausal osteoporosis by reducing blood glucose, lowering blood lipids, and alleviating oxidative stress	Xiao et al. 2022
		17β‐estradiol (E2), L‐ascorbic acid (VitC)	MG‐63 cells	100 μg/mL, 48 h	Enhances the efficacy of E2 in the treatment of postmenopausal osteoporosis	Zhou et al. 2022
10	Ostreidae, Ostrea	Powdered oyster shell calcium (POS‐Ca)	VitaD‐model	0.44% Ca, 28 days	Enhances calcium bioavailability, supports calcium homeostasis and bone mineralization	Tsugawa et al. 1995
		Calcium, amino acids and trace elements	GIO model	227.5 mg/kg/day, 10 weeks	Inhibits bone resorption, promotes bone formation, modulates gut microbiota	Shi et al. 2021
11	Rutaceae, Zanthoxylum	α‐linolenic acid, palmitoleic acid, oleic acid, linoleic acid	OVX model	0.57, 1.73, 3.46 mL/kg; 12 weeks	Inhibits inflammatory response and osteoclastogenesis in osteoporotic rats	Li 2022
		Polyunsaturated fatty acids (PUFAs)	RAW264.7 cells	0.005%–0.04% (*v*/*v*), 48–72 h	Inhibits osteoclastogenesis and bone resorption	He et al. 2022
12	Solanaceae, Lycium	*Lycium barbarum* polysaccharide	Zebrafish larvae, adult zebrafish	Larvae: 10, 100, 500 μg/mL, 6 days; Adults: 1, 5 μg/mL, 30 days	Promotes osteogenesis, enhances bone mineralization, inhibits osteoclast activation, and balances bone metabolism	Zheng et al. 2023
13	Polyporaceae, Poria	Hydroethanolic extract	OVX model	10, 20 mg/kg/day, 4 weeks	Inhibits osteoclastogenesis and bone resorption, reduces marrow adiposity, and ameliorated estrogen‐deficiency‐induced bone loss	Hwang et al. 2020
14	Campanulaceae, Platycodon	Platycodin D	C3H10T1/2 cells	0.001, 0.01, 0.1, 1 μmol/L; 5–10 days	Promotes osteoblast differentiation and mineralization by activating SIRT1/β‐catenin signaling via GSK3β inhibition	Han et al. 2019
		Deapioplatycoside E, platycoside E, deapioplatycodin D3, platycodin D2, D3, polygalacin D2	C2C12 cells	1, 2, and 4 μg/mL, 2–4 days	Stimulates osteoblast differentiation through p38 MAPK‐ and ERK‐dependent RUNX2 activation	Jeong et al. 2010
15	Zingiberaceae, Alpinia	Galangin	GIOP model, BMSCs cells	40 mg/kg/day, 8 weeks; 5, 10, 20 μmol/L	Ameliorates glucocorticoid‐induced osteoporosis	Zeng et al. 2023
16	Fabaceae, Glycine	Isoflavones, γ‐aminobutyric acid	Primary rat osteoblasts	1 × 10^−4^ g/L, 4 days	Improves bone formation and anti‐osteoporosis activity in vitro	Zhang et al. 2024
		Isoflavones	C3H10T1/2 cells	10, 100, 1000 μg/L, 24–72 h	Inhibits mesenchymal stem cell proliferation and promotes osteogenic differentiation	Ji and Li 2018
17	Asteraceae, Cichorium	Inulin, gallic acid, cinnamic acid, naringin, myricetin, apigenin, phenolics, flavonoids, alkaloids	GIO model	ACE (100 mg/kg/day), EPE (100 mg/kg/day), or ACE/EPE MIX (80/20 mg/kg/day), 8 weeks	Attenuates Dex‐induced osteoporosis by enhancing bone formation, reducing bone resorption, suppressing oxidative stress and inflammation, and modulating Nrf2/HO‐1 and RANK/RANKL/OPG pathways	Saleh et al. 2024
18	Asparagaceae, Polygonatum	PSAE	GIOP model	200, 400 mg/kg, 12 weeks	Inhibits RANKL‐induced osteoclast differentiation via suppression of ROS/MAPK/NF‐κB/NFATc1 signaling and enhances osteoblast function	Zhou 2024
19	Fabaceae, Pueraria	Puerarin (PUE)	OVX model	15, 30 mg/kg/day, 7 weeks	Improves bone microstructure and biomechanical properties	Zhou et al. 2025
20	Pedaliaceae, Sesamum	Sesamin, Sesamolin, Sesamol	CP‐induced model	3 mL/kg/day, 15 days	Improved bone microstructure and biomechanical properties	Yu and Zhang 2015
21	Rosaceae, Rubus	Fupenzi ketone	C3H10T1/2 cells	10–100 μg/mL	Enhances osteogenic differentiation of C3H10T1/2 stem cells and may facilitate bone formation independent of adipocyte differentiation	Takata and Morimoto 2014
22	Lamiaceae, Agastache	Patchouli aqueous extract	OVX model	100, 300 mg/kg/day, 5 weeks	Inhibits osteoclast differentiation and function by suppressing RANKL‐induced MAPKs and NF‐κB signaling, thereby reducing c‐Fos and NFATc1 expression	Jang et al. 2023
23	Apiaceae, Angelica	Angelica sinensis polysaccharide (s)	OVX model	200, 400 mg/kg	Exhibits therapeutic effects against osteoporosis in ovariectomized rats	Zheng 2015
24	Zingiberaceae, Curcuma	Curcumin	Osteoclast	5, 10, 20 μmol/L	Inhibits LPS‐induced osteoclast differentiation in RAW264.7 cells, possibly via suppression of p53/SLC7A11/GPX4 signaling	Li et al. 2024
25	Piperaceae, Piper	Piperlongumine	BMDMs cells	0.25, 0.5, 1, 2, 5, 10, 25, 50, 100 μmol/L	Inhibits RANKL‐induced NFATc1 expression	Huang and Wang 2023
26	Campanulaceae, Codonopsis	*Codonopsis pilosula* polysaccharide (CPS)	OVX model, BMSCs cells	100, 200, 400 mg/kg/day; 25, 50, 100 μg/mL	Serves as a functional food for bone health	Liu et al. 2023
27	Araliaceae, Panax	50% Ethanolic Extract	zebrafish model	2.5, 5, 10 μg/mL	Promotes bone formation and inhibit bone resorption	Qiu et al. 2023
28	Orobanchaceae, Cistanche	Cistanche Extract	OVX model	0.5 mg/kg/day	Excellent calcium supplements for the treatment of osteoporosis caused by OVX	Chu and Wu 2023
		Dendrobium officinale extract	OVX model	0.35, 0.70, 1.05 g/kg	Promotes bone formation and resist osteoporosis caused by OVX	Wang et al. 2021
29	Fabaceae, Astragalus	Astragaloside IV	OVX model	40 mg/kg/day	Improvement of bone properties and colonic mucosa in OVX rats, via modulation of gut microbiota composition	Wang et al. 2025
30	Polyporaceae, Ganoderma	Ganoderic Acid A, GA‐A	OVX model	In vivo: 5, 10, 20 mg/kg; In vitro: 5, 20, 80 μmol/L	Anti‐osteoporosis effect by promoting osteoblast differentiation and bone formation via activating PIK3CA/Akt and inhibiting TWIST1	Zhao et al. 2025
31	Cornaceae, Cornus	3‐Amino‐10‐hydroxyl‐evodiamine (E2)	OVX model	In vitro: 2.5, 5, 10 μmol/L; in vivo: 1 mg/kg, 4 weeks	Anti‐osteoporotic action via suppression of osteoclast differentiation through inhibition of RANKL/NF‐κB/MAPK/NFATc1 signaling	Liu, Shen, et al. 2024
32	Orchidaceae, Gastrodia	Gastrodin	Osteoblasts	5 μmol/L	Potential to improve osteointegration in diabetic implants by enhancing antioxidant levels and reducing oxidative stress	Li et al. 2023
33	Eucommiaceae, Eucommia	Astragalin, Baicalein	MC3T3‐E1 S14	7.5, 40, 807.5 mg/L	Promotes proliferation of MC3T3‐E1 cells by up‐regulating Osterix and OPG/RANKL ratios	Xiao et al. 2023
34	Orobanchaceae, Rehmannia	Steamed Rehmannia Extract	GIOP model	In vivo: 1, 2, 4 g/kg; In vitro: 0.2, 1, 5 mg/L	Promotes bone formation through modulation of sex hormone levels, targeting steroid biosynthesis pathway to upregulate CYP17A1/CYP19A1 and downregulate HSD11B1	Xiao et al. 2019

In terms of model types, level 2 plants mainly adopted the ovarian resection (OVX) model and the glucocorticoid induction (GIO) model. From the perspective of mechanism of action, it can be summarized into the following categories: hawthorn, purslane, licorice, nutmeg, cinnamon, peppercorn, poria, chicory, astragalus, dogwood, etc., and its active ingredients regulate the OPG/RANKL/RANK axis and inhibit osteoclast differentiation by down‐regulating NFATc1, c‐Fos, TRAP, CTSK, MMP‐9 and other osteoclast‐specific genesInhibits osteoclast production and bone resorption. Pueraria kudzu, platycodon root, *eucommia ulmoides* , yam, and so forth, by activating Wnt/β‐catenin, BMP/Smad or p38 MAPK/ERK signaling pathways, up‐regulating Runx2 and Osterix, promoting osteoblast differentiation and bone formation. Sea buckthorn, wolfberry, chicory, yellow essence, gastrodia, rehmannia, and so forth, reduce oxidative stress and inflammatory response, and protect bone cells by activating Nrf2/HO‐1, inhibiting NF‐κB and MAPK signals. Galangal, Ganoderma lucidum, astragalus, and so forth, regulate autophagy flux through AMPK/mTOR, PKA/CREB or PI3K/Akt pathways to promote osteoblast survival. Oysters, astragalus, cistanche, and so forth, play a role by regulating the structure of intestinal flora, mineral metabolism, increasing the production of short‐chain fatty acids, or supplementing trace elements such as calcium.

Level 3 refers to at least one independent randomized controlled trial (RCT) support, but there are limitations such as small sample size, short follow‐up time, or unclear risk of bias; or evidence of observational human studies such as prospective cohorts.

The evidence for human studies of Ejiao is relatively rich. One scoping review included 22 studies (5 Ejiao alone and 17 compounds). Ejiao alone animal experiments showed that it could enhance osteogenic differentiation, and clinical studies suggested that the combination of calcium was more effective. An RCT of 128 patients showed that Ejiao Strong Bone Oral Solution combined with calcitriol was superior to calcitriol alone in bone density and pain relief. Meta‐analysis showed that Ejiao‐containing compound preparations improved bone mineral density and had low adverse effects (Ekeuku et al. 2025). However, the clinical evidence of Ejiao alone is limited, and it is mostly used in compound form. Preliminary human studies of olive extract showed that 250 mg daily of olive polyphenol extract did not report a clear improvement in bone density (Filip et al. 2015). Animal experiments suggest that the osteoprotective effect is model‐dependent. The evidence is currently mainly from small studies and needs further validation. A 4‐month RCT of ginger extract reported that ginger combined with curcumin supplementation (500 mg/day each) improved osteocalcin, alkaline phosphatase, hs‐CRP, and SOD levels in postmenopausal women with osteoporosis (Salekzamani et al. 2023; Aghamohammadi et al. 2020). There are currently no human studies of ginger extract alone, and its effects cannot be fully attributed to ginger itself.

In summary, the three levels of FMH show initial potential for human application, but the clinical evidence for individual components is insufficient, and most studies have limitations such as small sample sizes, complex interventions, or incomplete interventions. In the future, multi‐model in vivo experiments and larger‐scale, longer follow‐up, and standardized randomized controlled trials of interventions are needed to confirm its clinical efficacy and safety.

## 5 Discussion and Expectation

FMH mainly slows down osteoporosis by reducing oxidative stress, estrogen effects, and regulating autophagy and gut microbial metabolism. However, the multicomponent nature of FMH makes its mechanism difficult to elucidate and its reproducibility is limited. Different studies differed greatly in terms of dosage, type of administration (extracts and monomer compounds), route of administration and duration of exposure, and the influencing factors included extract standardization, model differences (rodents and zebrafish; OVX and GIO), bioavailability and nonlinear dose–response relationship. The anti‐osteoporosis effects of FMH plants and their active ingredients often exhibit nonlinear dose‐effect characteristics. In most studies, low doses only produced limited regulatory effects, while medium doses improved bone density, bone microstructure and bone metabolism more stably. High doses do not produce optimal effects, even lower than moderate dose levels. This phenomenon is in line with the concept of “hormesis”: low doses stimulate the defense pathway of Nrf2, SIRT1, PI3K/Akt, Wnt/β‐catenin, while too high doses may lead to effect platforming or decline due to redox imbalance, receptor saturation, or metabolic burden. Taking flavonoids as examples, the dose response of quercetin and kaempferol has obvious biphasic. Resveratrol and curcumin also exhibit similar characteristics in bone metabolism. Therefore, the “optimal dose” of FMH should be understood as a therapeutic window rather than a linear escalation relationship. Although multiple FMH compounds show osteoprotective properties, long‐term safety data are limited. Icariin and related isoprenyl flavonoids have estrogen‐like activity, and its metabolite icariin can stimulate the proliferation of estrogen receptor‐positive breast cancer cells at low concentrations. Therefore, when used for long‐term prophylaxis in women with a history of breast disease, tissue selectivity safety (including uterine and breast pathology and long‐term carcinogenicity studies) needs to be carefully evaluated. Although lignans in sesame seeds have antioxidant and hepatoprotective effects, data on chronic high‐dose exposure and standard toxicological endpoints are still lacking, and liver function should be monitored with long‐term use of high‐dose preparations.

Standard drugs (bisphosphonates, denosumab, SERMs, teriparatide, etc.) can reduce the risk of fractures, but there are limitations such as atypical femoral fractures, mandibular osteonecrosis, and rebound after drug discontinuation. Compared with standard anti‐osteoporotic drugs, there was no fracture endpoint RCT in FMH resources, and the improvement of bone mineral density and the regulation of bone turnover markers were lower than those of standard drugs, which could not replace standard treatment. Standard drugs are single‐target, and FMH is multi‐target synergy, which is more suitable for long‐term prevention and early intervention. FMH is generally safe at recommended doses, but the long‐term safety of phytoestrogens on the mammary/endometrium is unknown; Some studies suggest that FMH may be used as an adjunct to standard treatment (e.g., total flavonoids combined with alendronate). Standard drugs are based on grade 3 or higher evidence, and FMH is predominantly preclinical or small‐sample exploratory evidence and is currently suitable for positioning as a functional food or dietary supplement. In the future, multicenter RCTs with bone mineral density and fracture as endpoints need to be carried out, and long‐term safety and drug interactions should be systematically evaluated.

It is important to note that this review strictly followed the established inclusion criteria for the evidence (PubMed and CNKI databases, last 15 years, in vivo/in vitro/randomized controlled trials). Therefore, although four substances have been officially added to the 2024 National List of Medicinal and Food Homologies (*Rehmannia glutinosa*, *Ophiopogon japonicus* , and *Asparagus cochinchinensis*) and *Citrus grandis* “Tomentosa,” but no experimental studies of these four plants directly targeting osteoporosis as a single FMH intervention were found to meet the inclusion criteria for this review. Nevertheless, the traditional application of these four substances and their active ingredients (e.g., catala alcohol in Rehmannia, Ophiopogon saponin D in Ophiopogon and naringin in Orange Red) have been reported anti‐osteoporosis, or their use as components of compound formulations, suggesting that these four FMH plants have osteoprotective potential. In the future, there is an urgent need for direct in vitro and in vitro studies of these four plants as whole FMH substances to fill the current knowledge gap.

## 6 Conclusions

Osteoporosis is a metabolic bone disease that primarily affects postmenopausal women and the elderly. This review systematically summarizes the evidence from preclinical studies on FMH plants and their active ingredients. In terms of mechanism, it mainly has estrogen‐like effects, inhibits oxidative stress, regulates intestinal flora, regulates autophagy, and plays a role in osteoprotective effects. Based on the three‐level evidence classification, 24 plants had only a single animal/in vitro study (level 1), 34 had consistent results from multiple independent animal studies (level 2), and a few (Ejiao, olive, ginger) had preliminary human trials, but there were obvious limitations. The current evidence is mainly preclinical studies, and there is a lack of high‐quality randomized controlled trials, and issues such as extract standardization, bioavailability, long‐term safety, and drug interactions have not been addressed. In summary, FMH resources have a pharmacological basis in osteoporosis prevention and adjuvant treatment, but the efficacy should not be exaggerated, and should be positioned as an adjunctive strategy for long‐term prevention and early intervention. In the future, clinical trials with fractures as endpoints need to focus on dose optimization, chronic toxicity assessment, and fractures.

## Author Contributions

**Long Zhao:** methodology, writing – review and editing, writing – original draft, conceptualization. **Rui‐ting Li:** investigation, writing – original draft. **Xiao‐feng Shi:** writing – review and editing, conceptualization, supervision, funding acquisition, resources, project administration. **Qu‐huan Ma:** conceptualization, writing – original draft. **Xiao‐li Dong:** investigation, writing – original draft.

## Funding

This work was supported by the Lanzhou Science and Technology Bureau [2024‐02‐07].

## Conflicts of Interest

The authors declare no conflicts of interest.

## Data Availability

No data were used to support this study.
